# *Sinorhizobium fredii* HH103 RirA Is Required for Oxidative Stress Resistance and Efficient Symbiosis with Soybean

**DOI:** 10.3390/ijms20030787

**Published:** 2019-02-12

**Authors:** Juan Carlos Crespo-Rivas, Pilar Navarro-Gómez, Cynthia Alias-Villegas, Jie Shi, Tao Zhen, Yanbo Niu, Virginia Cuéllar, Javier Moreno, Teresa Cubo, José María Vinardell, José Enrique Ruiz-Sainz, Sebastián Acosta-Jurado, María José Soto

**Affiliations:** 1 Departamento de Microbiología, Facultad de Biología, Universidad de Sevilla, Avda. Reina Mercedes 6, 41012 Sevilla, Spain; jccresporivas@gmail.com (J.C.C.-R.); pnavarro2@us.es (P.N.-G.); calias@us.es (C.A.-V.); cubo@us.es (T.C.); jvinar@us.es (J.M.V.); rsainz@us.es (J.E.R.-S.); 2 Daqing Branch of Heilongjiang Academy of Sciences, Daqing 163000, China; shijie0456@163.com; 3 Institute of Microbiology, Heilongjiang Academy of Sciences, Harbin 150001, China; zhentao1212@126.com (T.Z.); lniuyanbo@163.com (Y.N.); 4 Departamento de Microbiología del Suelo y Sistemas Simbióticos, Estación Experimental del Zaidín, CSIC, c/ Profesor Albareda 1, 18008 Granada, Spain; virginia.cuellar@eez.csic.es; 5 Departamento de Biología Celular, Facultad de Biología, Universidad de Sevilla, Avda. Reina Mercedes 6, 41012 Sevilla, Spain; onorato@us.es

**Keywords:** iron, *Rhizobium*, regulation, siderophore, nitrogen-fixation, plant–bacteria interaction

## Abstract

Members of *Rhizobiaceae* contain a homologue of the iron-responsive regulatory protein RirA. In different bacteria, RirA acts as a repressor of iron uptake systems under iron-replete conditions and contributes to ameliorate cell damage during oxidative stress. In *Rhizobium leguminosarum* and *Sinorhizobium meliloti*, mutations in *rirA* do not impair symbiotic nitrogen fixation. In this study, a *rirA* mutant of broad host range *S. fredii* HH103 has been constructed (SVQ780) and its free-living and symbiotic phenotypes evaluated. No production of siderophores could be detected in either the wild-type or SVQ780. The *rirA* mutant exhibited a growth advantage under iron-deficient conditions and hypersensitivity to hydrogen peroxide in iron-rich medium. Transcription of *rirA* in HH103 is subject to autoregulation and inactivation of the gene upregulates *fbpA*, a gene putatively involved in iron transport. The *S. fredii rirA* mutant was able to nodulate soybean plants, but symbiotic nitrogen fixation was impaired. Nodules induced by the mutant were poorly infected compared to those induced by the wild-type. Genetic complementation reversed the mutant’s hypersensitivity to H_2_O_2_, expression of *fbpA*, and symbiotic deficiency in soybean plants. This is the first report that demonstrates a role for RirA in the *Rhizobium*-legume symbiosis.

## 1. Introduction

As for almost all living organisms, iron is an essential nutrient for bacteria but in excess it becomes toxic. This active redox metal forms part of the prosthetic group (heme or iron–sulfur clusters) of many proteins that participate in important metabolic processes such as respiration, central metabolism, redox stress resistance, or nitrogen fixation. Although it is not a rare element in the Earth’s crust, iron is not readily bioavailable in most environments. Under aerobic conditions and at neutral pH values, the concentration of soluble iron compounds is too low to fulfill the cell needs. Bacteria circumvent this problem with high-affinity uptake systems whose expression is triggered under iron-deficient conditions [1]. On the other hand, the redox properties of iron are responsible for the generation of highly toxic free radicals via the Fenton reaction. Reactive oxygen species (ROS) such as H_2_O_2_ generated during aerobic metabolism in combination with ferrous iron leads to the production of hydroxyl radicals (OH^•^), which are extremely reactive oxidants capable of damaging DNA, proteins, and lipids [2]. During oxidative stress, i.e., an increased ROS production that cannot be balanced by antioxidants, toxicity caused by iron is more critical. Superoxide (O_2_^•-^) and hydrogen peroxide (H_2_O_2_) can react with iron present in the prosthetic group of metalloproteins, resulting in enzyme inhibition and the release of free iron, which could then promote the Fenton reaction [3]. Therefore, bacteria require mechanisms for iron homeostasis that allow them to maintain sufficient levels of the metal to support vital metabolic processes and at the same time prevent toxicity.

Iron homeostasis is achieved by controlling the expression of proteins involved in iron uptake, storage, consumption, and export. When intracellular levels of iron are insufficient, many bacteria produce siderophores, i.e., water soluble Fe^3+^ chelating compounds, and express several high-affinity uptake systems to acquire iron from the environment [4]. Under iron sufficient conditions, expression of iron uptake systems is repressed and iron is incorporated into metalloproteins. Excessive iron is accumulated in a non-reactive form in iron storage proteins and/or expelled out of the cell by iron efflux transporters to mitigate cytotoxic effects [5,6,7]. All of these processes are coordinately controlled through the activity of iron response regulatory proteins.

In many bacteria, including *Escherichia coli*, the major iron response regulator is Fur (Ferric Uptake Regulator) [1,8,9,10]. Fur is a transcriptional regulator that senses and responds to iron by directly binding this metal [10]. In the presence of certain levels of iron, the Fe^2+^-Fur complex binds to DNA sequences called Fur boxes and represses the transcription of iron uptake systems, preventing iron overload toxicity [1]. Remarkably, iron-responsive regulation differs from the Fur paradigm in rhizobia, i.e., soil-dwelling bacteria that are known for their ability to establish a symbiotic relationship into nitrogen-fixing nodules on the roots of leguminous plants [11]. Symbiotic nitrogen fixation is highly dependent on iron because different proteins involved in the process require the metal to be functional. This is the case of the nitrogenase complex responsible for the nitrogen fixation reaction, or the abundant nodule protein leghemoglobin that buffers oxygen levels to avoid inactivation of nitrogenase activity. Although a Fur-like protein is present in most rhizobia, functional studies performed on the *fur* genes of *Sinorhizobium (Ensifer) meliloti* and *Rhizobium leguminosarum* revealed that the encoded proteins are responsive to manganese and not to iron, which is the reason why they were renamed as Mur [12,13,14]. Instead, iron-responsive regulation in rhizobia is mediated by the Irr and RirA regulatory proteins that sense iron availability indirectly [15,16,17].

The Irr (Iron Responsive Regulator) protein belongs to the Fur superfamily. Described initially in the soybean symbiont *Bradyrhizobium japonicum* [18], Irr occurs in all rhizobia and is the only iron responsive regulator in *Bradyrhizobiaceae*. Under iron-deficient conditions, Irr usually represses genes encoding proteins that function under iron-sufficient conditions (i.e., proteins containing iron, heme and iron–sulfur cluster biosynthetic proteins). Upon sensing iron indirectly through the status of heme biosynthesis, Irr is either degraded (as occurs in *B. japonicum*) or becomes inactive (as is the case of *R. leguminosarum*), which allows transcription of Irr-repressed genes [17].

In rhizobia distinct from *Bradyrhizobiaceae*, iron homeostasis is achieved with the participation of a second regulatory protein, RirA (Rhizobial Iron Regulator A). This regulator, which is found exclusively in alphaproteobacteria, is present in *Rhizobium*, *Sinorhizobium* (*Ensifer*), and *Mesorhizobium* spp., but also in the phytopathogen *Agrobacterium tumefaciens* and in the animal pathogens *Brucella* and *Bartonella* [14,17]. RirA is an iron–sulfur protein that belongs to the Rrf2 family of putative transcription regulators. Under iron-replete conditions, this regulator acts as a repressor of many iron-responsive genes by binding to Iron-Responsive Operator sequences (5’-TGA-(N9)-TCA-3’) known as IRO boxes [14,19]. A recent study has demonstrated that *R. leguminosarum* RirA contains a [4Fe–4S] cluster and that the holoprotein ([4Fe–4S]RirA) binds to an IRO box sequence [20]. Interestingly, the [4Fe–4S] cluster is sensitive to iron levels but also to O_2_, with low iron concentrations and aerobic conditions promoting the conversion of [4Fe–4S] cluster to [2Fe–2S] and subsequent cluster loss, which relieves gene repression [20]. Therefore, RirA seems to act as an iron sensor via iron–sulfur cluster availability.

In rhizobia, RirA has been functionally characterized only in *R. leguminosarum* and *S. meliloti*. In *R. leguminosarum*, RirA regulates several iron-responsive genes such as genes for the synthesis and uptake of the siderophore vicibactin (*vbs*), genes involved in heme uptake (*hmu* and *tonB*), genes for the synthesis of iron–sulfur clusters (*suf*), the *irrA* gene as well as its own expression [21,22]. In *S. meliloti*, the RirA regulon comprises several iron-responsive genes, which include genes involved in iron uptake, energy metabolism, exopolysaccharide synthesis or iron storage, but also genes that are not iron-responsive (e.g., *suf*) [23,24,25]. Several free-living phenotypes have been associated with *rirA* mutants. Thus, inactivation of *rirA* leads to: (i) constitutive expression of siderophores in *R. leguminosarum*, *S. meliloti* and in *A. tumefaciens*, (ii) species-specific growth phenotypes which can be iron-dependent or -independent, (iii) higher sensitivity to oxidants in *S. meliloti* and in *A. tumefaciens*, and (iv) decreased ability to cope with cobalt and nickel toxicity in *A. tumefaciens* [21,23,24,26,27,28]. Hypersensitivity to peroxides and metals in *rirA* mutants was explained by the derepression of iron uptake systems and the consequent iron overload in the cell, which promotes the generation of ROS via Fenton chemistry.

The role of RirA in the establishment of plant–bacteria interactions has also been investigated. Loss of function of RirA in *R. leguminosarum* or *S. meliloti* does not affect symbiotic nitrogen fixation [21,23,24]. In *A. tumefaciens*, RirA was found to be important for tumor formation on tobacco leaf pieces [26] but not on potato disks [27]. The defect in tumorigenesis on tobacco leaves was associated with increased sensitivity to oxidants and reduced virulence gene expression exhibited by the *rirA* mutant.

*S. fredii* HH103 is a fast-growing rhizobial strain, which was isolated from a Chinese soil from the Hubei Province. HH103 is able to nodulate American and Asiatic varieties of soybean, as well as many other different legumes that form determinate or indeterminate nodules [29,30]. Iron homeostasis in broad host range rhizobia has not been investigated yet. The genome sequence of *S. fredii* HH103 reveals the presence of putative iron-responsive regulatory genes, including a *rirA* homologue [31,32]. With the aim of gaining insights into iron-mediated responses in HH103, in this study, we have investigated the role of RirA during free-living growth and in symbiosis with soybean. Our data demonstrate that, in *S. fredii* HH103, RirA is important for oxidative stress resistance and for effective symbiosis with soybean plants.

## 2. Results

### 2.1. The Lack of RirA in S. fredii HH103 Confers a Siderophore-Independent Growth Advantage under Low Iron Conditions

*S. fredii* HH103 encodes homologues of Fur (SFHH103_03131), Irr (SFHH103_03814), and RirA (SFHH103_00388). The chromosomal *SFHH103_00388* gene encodes a 154 amino acid-long protein annotated as an iron-responsive regulator of the Rrf2 family of transcriptional regulators. The predicted protein, which is identical to one encoded by *S. fredii* NGR234, exhibits high sequence similarity to the RirA proteins of *S. meliloti*, *A. tumefaciens* and *R. leguminosarum* bv. viciae (93.5%, 83.4%, and 79.4% identity, respectively), and conserves the three Cys residues predicted to ligate iron–sulfur clusters in Rrf2 family regulators [20] (Figure S1). The genetic context of the *rirA* gene is similar in HH103 and in *S. meliloti*. In the two rhizobia, the gene is flanked downstream by a cluster of *dpp* genes involved in the uptake of dipeptides and a heme precursor [33], and upstream by the *fbpA* gene that potentially codes for a periplasmic ferric binding protein, which is regulated by RirA in *S. meliloti* [23].

In order to investigate the role of the *rirA* homologue in HH103, mutant SVQ780 was constructed by inserting a *lacZ*Δp-Gm^R^ cassette into the coding sequence of this gene (see Material and Methods). A battery of free-living phenotypes that have been associated with *rirA* mutants were evaluated such as siderophore production, growth in media with different iron content, resistance to H_2_O_2_, and expression of iron-responsive genes.

Under our experimental conditions, Chrome Azurol S (CAS) assays performed using cultures of the wild-type strain SVQ269 (HH103-Rif^R^) grown in iron-deficient media failed to show any siderophore activity. In fact, CAS assays performed on forty *S. fredii* strains isolated from different locations in China only showed activity in two of them (strains S2 and S5; Figure S2), suggesting that siderophore production is a rare attribute in Chinese *S. fredii* strains. RirA loss-of-function leads to overexpression of siderophore biosynthesis and transport genes in *R. leguminosarum*, *S. meliloti* and *A. tumefaciens* [22,23,24,26]. However, no CAS activity was detected upon inactivation of the *rirA* homologue in HH103 (Figure S2c), indicating that this strain does not acquire iron via CAS-detected siderophore production.

**Figure 1:**
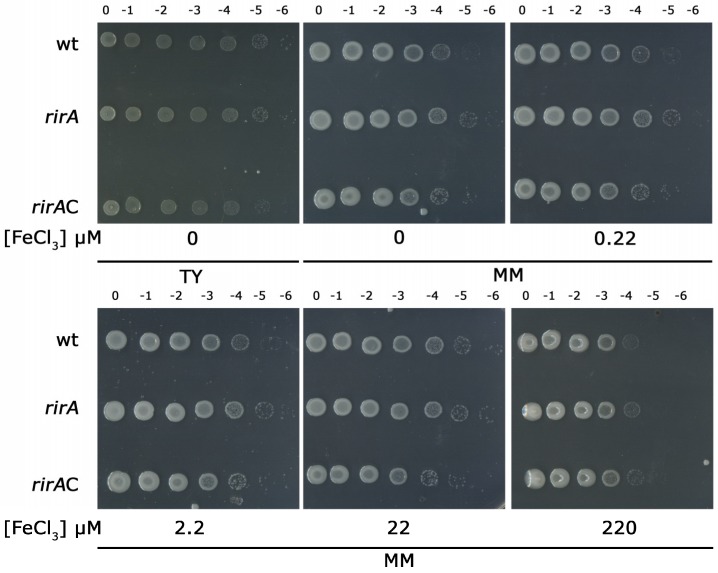
Growth of *S. fredii* wild-type strain SVQ269 (wt), *rirA* mutant SVQ780 (*rirA*) and complemented strain SVQ780C (*rirA*C) on tryptone-yeast extract (TY) and minimal medium (MM) agar plates in the absence or presence of different concentrations of FeCl_3_ as indicated. Tenfold serial dilutions of bacterial cultures (10^9^ cells/mL) are marked above each column. A representative example of at least two experiments is shown.

Depending on the bacterial species, inactivation of *rirA* results in different growth phenotypes ranging from iron-dependent or -independent reduced growth to no effect at all [21,23,27]. In our study, we evaluated the growth rates of *S. fredii* wild-type and *rirA* mutant strains in minimal medium (MM) agar plates supplemented with different concentrations of FeCl_3_ as the added iron source, as well as in un-supplemented MM with ambient iron levels. As shown in Figure 1, the *rirA* mutant exhibited similar growth capacities in un-supplemented MM and in MM supplemented with 0.22, 2.2 and 22 µM FeCl_3_. The wild-type and the *rirA* mutant exhibited similar growth rates on tryptone-yeast extract (TY) medium. Likewise, no differences were detected between the two strains on MM plates supplemented with 22 µM FeCl_3_, which is the optimum concentration of iron for the wild-type strain under the conditions tested. Interestingly, on MM containing lower concentrations of iron (i.e., un-supplemented and supplemented with 0.22 and 2.2 µM FeCl_3_) the *rirA* mutant showed a slight advantage in growth with respect to SVQ269 and the complemented strain SVQ780C. The low iron availability-dependent growth advantage associated with the *rirA* mutant might be caused by derepression of as yet uncharacterized high-affinity iron uptake systems. In MM with the highest iron content (220 µM FeCl_3_), all three strains exhibited similar growth reductions compared with MM containing 22 µM FeCl_3_, suggesting probable iron toxicity.

### 2.2. RirA Plays A Role in Oxidative Stress Resistance in HH103

It has been shown that *rirA* mutants of *S. meliloti* and *A. tumefaciens* are more sensitive to oxidative stress than their corresponding wild-type strains [23,26,27]. Therefore, we decided to test whether inactivation of *rirA* in HH103 alters the bacterial resistance to hydrogen peroxide.

**Figure 2:**
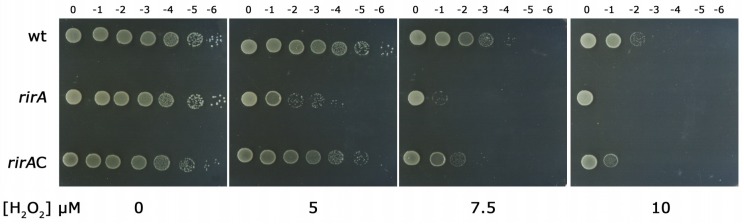
Growth of *S. fredii* wild-type strain SVQ269 (wt), *rirA* mutant SVQ780 (*rirA*) and complemented strain SVQ780C (*rirA*C) on TY in the absence or presence of different concentrations of H_2_O_2_. Tenfold serial dilutions of bacterial cultures (10^9^ cells/mL) are marked above each column. A representative example of at least two experiments is shown.

During these experiments, we noticed that HH103 was much more sensitive to H_2_O_2_ than *S. meliloti* or *A. tumefaciens*. Thus, approximately only 1 out of 10^7^ cells survived after exposing SVQ269 cultures to 10 mM H_2_O_2_ for only 1 h. When SVQ269 cultures were exposed to a 100-fold lower concentration of H_2_O_2_ (100 µM), the survival ratio of the wild-type strain was slightly reduced to 71%. Under the same conditions, the viability of *rirA* mutant cells was extremely low (only 20 out of every 10^7^ cells could survive after treatment with 100 µM H_2_O_2_). The higher sensitivity of the *rirA* mutant to H_2_O_2_ compared to the wild-type strain was confirmed by growing serial dilutions of cells on TY plates containing different concentrations of the oxidant (Figure 2).

No differences in growth were observed for the wild-type and mutant strains on TY plates in the absence of H_2_O_2_. While viability of SVQ269 remained unchanged in the presence of 5 µM H_2_O_2_, survival of the *rirA* mutant was already severely affected. The presence of higher concentrations of H_2_O_2_ (7.5 and 10 µM) decreased the viability of the wild-type and especially that exhibited by the *rirA* mutant. The hypersensitive phenotype of SVQ780 could be reversed by introducing a functional *rirA* gene in *cis* (strain SVQ780C). These data indicate that inactivation of *rirA* in *S. fredii* HH103 results in reduced resistance to oxidative damage.

### 2.3. Role of RirA in Iron-Responsive Gene Expression

Inactivation of *rirA* in *R. leguminosarum*, *S. meliloti*, and *A. tumefaciens* causes upregulation of several genes whose transcription is normally reduced/repressed under iron-sufficient conditions. These include genes involved in the synthesis and uptake of siderophores, heme uptake, inorganic ferric ion transport, biosynthesis of Fe–S clusters, and the regulatory *irr* and *rirA* genes [21,22,23,24,26,27].

**Figure 3:**
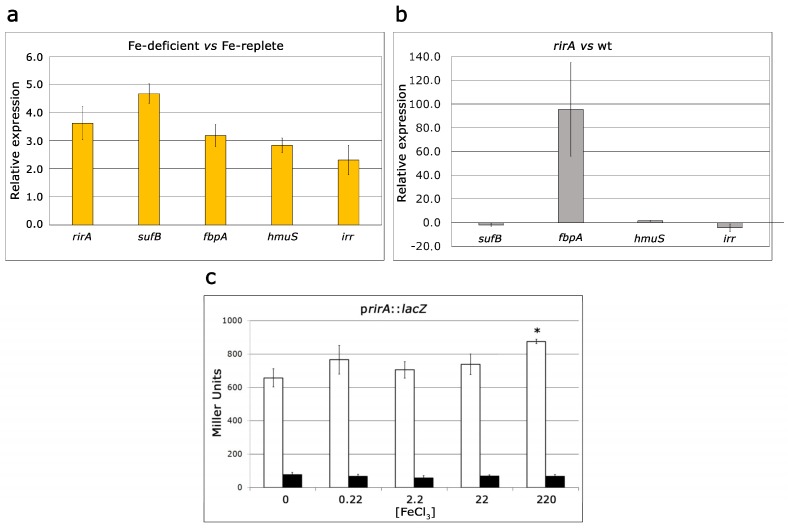
Gene expression analyses of iron-related genes in *S. fredii*. (**a**) Relative expression of *rirA*, *sufB*, *fbpA*, *hmuS* and *irr* genes in the wild-type strain SVQ269 of *S. fredii* as determined by Reverse Transcription-quantitative Polymerase Chain Reaction (RT-qPCR). The relative expression was calculated as the fold change between growth in iron-deficient (0.22 µM FeCl_3_) and iron-replete (22 µM FeCl_3_) media; (**b**) relative expression of iron-related genes in *rirA* mutant versus wild-type strain after growth in iron-sufficient (22 µM FeCl3) medium as determined by RT-qPCR; (**c**) transcriptional activity of *rirA* in the SVQ780 mutant (white bars) and complemented strain (SVQ780C) (black bars) after growth in media with different iron concentration as determined by β-galactosidase activity. Results are averages from at least two independent biological experiments with two technical replicates. Error bars indicate standard error. The asterisk indicates significant difference with respect to data obtained in cells grown in un-supplemented media according to ANOVA test (*p* < 0.05).

The expression levels of potentially iron-responsive genes were determined by Reverse Transcription-quantitative Polymerase Chain Reaction (RT-qPCR) in the wild-type strain SVQ269 after growth in iron-deficient or iron-replete media. Based on the bacterial growth shown in Figure 1, MM supplemented with 0.22 and 22 µM FeCl_3_ were used as the iron-deficient and iron–replete conditions, respectively. The chosen genes comprise those putatively coding for the periplasmic binding protein of a ferric type ATP-Binding Cassette (ABC) transporter (*fbpA*), the ATPase component of a heme ABC transporter (*hmuS*), a protein involved in Fe-S cluster formation (*sufS*), the Irr homologue (SFHH103_03814), and *rirA*. As shown in Figure 3a, all five genes exhibited upregulation in wild-type cells grown in iron-deficient medium compared with cells grown in iron-replete medium.

To examine the effects of RirA on iron homeostasis, the transcript levels of *fbpA*, *hmuS*, *sufS* and *irr* were also determined by RT-*q*PCR in *rirA* mutant cells after growth in iron-sufficient (22 µM FeCl_3_) media (Figure 3b). Under these conditions, no differential expression was detected for *hmuS* between the mutant and the wild-type (1.5 ± 0.3 fold increase in *rirA* mutant versus wild-type strain). In the case of *sufB* and *irr*, only slight decreases in expression were observed in the mutant compared to the wild-type (2 ± 1.3 and 4.4 ± 3.3 fold decrease in *rirA* mutant versus wild-type strain for *sufB* and *irr*, respectively). In contrast, inactivation of *rirA* caused a clear upregulation of *fbpA* (95.4 ± 39.6 fold increase in *rirA* mutant versus wild-type strain). No differential expression for *fbpA* was detected between the wild-type and the complemented strain. This suggests that RirA represses transcription of *fbpA*, either directly or indirectly.

Since the promoterless *lacZ*-Gm^R^ cassette used to obtain the *rirA* mutant was inserted in the same transcriptional orientation as that of the mutated gene, *rirA* expression levels could be determined by measuring β-galactosidase activity in SVQ780 and SVQ780C strains grown in un-supplemented MM or in MM supplemented with different iron concentrations. As shown in Figure 3c, expression of *rirA* in the SVQ780 mutant increased slightly with increased iron content in the medium, exhibiting the lowest and highest expression levels in un-supplemented MM and in MM containing 220 µM FeCl_3_, respectively. These results are in agreement with data obtained in *R. leguminosarum* and in *A. tumefaciens rirA* mutants, in which *rirA* expression was downregulated under iron-deplete conditions [21,27]. In the complemented SVQ780C strain, expression of *rirA* was low irrespective of the concentration of iron in the medium, indicating an auto-regulatory role similar to that of RirA in *R. leguminosarum* and in *A. tumefaciens* [21,27].

### 2.4. Nitrogen-Fixing Symbiosis with Soybean Plants Is Impaired in an S. fredii HH103 rirA Mutant

**Figure 4:**
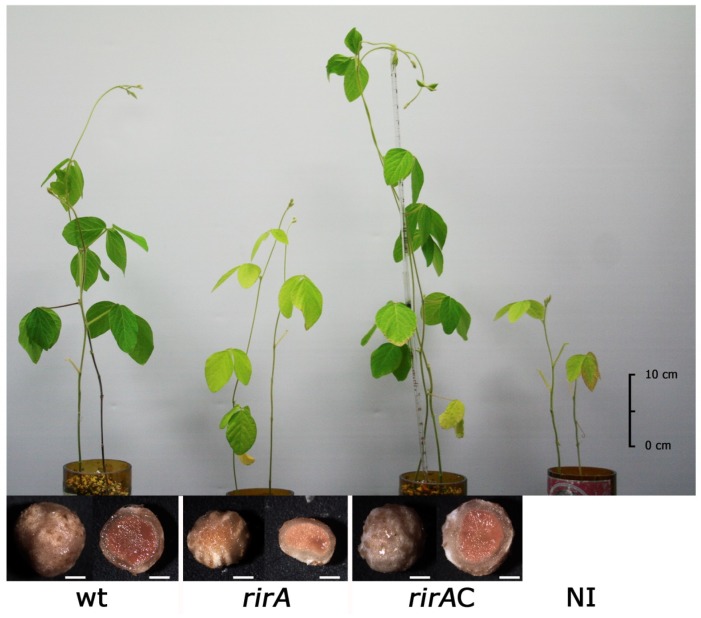
Symbiotic phenotype and nodule morphology of soybean plants inoculated with *S. fredii* SVQ269 (wt), its *rirA* derivative SVQ780 (*rirA*), and the complemented strain SVQ780C (*rirA*C). Non-inoculated plants are denoted as NI. Scale bars next to nodules correspond to 1 mm. A representative example of at least two experiments is shown.

Previous studies have shown that mutations in *rirA* in *R. leguminosarum* or in *S. meliloti* do not affect nodulation or symbiotic nitrogen fixation with their respective hosts (peas and vetch for *R. leguminosarum*, and alfalfa for *S. meliloti*) [21,23,24]. The symbiotic phenotype of the *S. fredii rirA* mutant was investigated in soybean (*Glycine max*) cv. Williams, which forms determinate nodules. The mutant was able to induce nodule formation on soybean roots. However, plants inoculated with the *rirA* mutant exhibited symptoms of impaired symbiotic effectiveness since they were smaller than plants inoculated with the wild-type strain and their leaves showed a chlorotic appearance similar to that exhibited by non-inoculated soybeans (Figure 4). These symptoms were suggestive of nitrogen deficiency.

**Figure 5:**
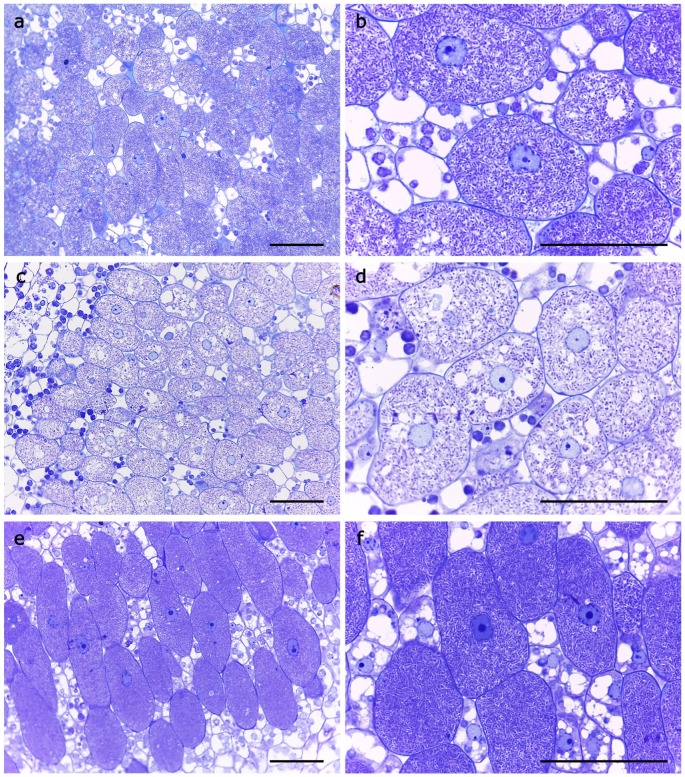
Optical microscopy of nodules induced in *G. max* cv. Williams by *S. fredii* wild-type strain SVQ269 (**a**–**b**); *rirA* mutant SVQ780 (**c**–**d**); and complemented strain SVQ780C (**e**–**f**). Bars correspond to 50 µm.

No significant differences were detected in the number of nodules developed in the roots of soybean plants inoculated with the wild-type or the *rirA* mutant. However, the nodules induced by the mutant exhibited a significant reduction in size and fresh weight and were not as red as those induced by the wild-type strain (Figure 4 and Table 1). Moreover, acetylene reduction assays (ARA) revealed that nitrogenase activity of the mutant was 5.6-fold reduced with respect to that of SVQ269 (Table 1). In order to better understand the symbiotic deficiency of the mutant, nodules induced by the wild-type and the *rirA* mutant were analyzed by light microscopy (Figure 5).

The results revealed that infected cells in nodules induced by the mutant contained fewer bacteria than those in nodules formed by the wild-type strain. The complemented strain SVQ780C regained the wild-type phenotype in symbiosis with soybean, including efficient nitrogen fixation (Figure 4 and Table 1) and degree of bacterial infection in nodule cells (Figure 5). Altogether, these data indicate that RirA is important for efficient symbiosis with soybean plants.

## 3. Discussion

In this work, we investigated the role of RirA, the rhizobial iron regulator, in *S. fredii* HH103. This study represents the first approach to tackle iron homeostasis in a broad-host range *Rhizobium*. Despite the pivotal role of iron in the establishment of efficient *Rhizobium*-legume symbiosis, studies dealing with how the microsymbiont controls the intracellular iron pools have only been performed in three rhizobia: *B. japonicum*, *R. leguminosarum* and *S. meliloti*. Whereas important insights have been obtained in *B. japonicum*, knowledge about iron homeostasis in *Rhizobiaceae* is limited. This is probably due to the fact that the different iron-related genes that have been characterized in *R. leguminosarum* and *S. meliloti*, including the iron regulators *irr* and *rirA*, are not essential for the formation of effective symbiosis with their respective host plants. Our study demonstrates that RirA is important for the symbiosis that *S. fredii* HH103 establishes with soybean.

*S. fredii* HH103 encodes homologues of three regulatory proteins that have been involved in iron homeostasis in different bacteria: Fur, Irr, and RirA. In *R. leguminosarum* and in *S. meliloti*, Irr and RirA participate in iron-dependent regulation in a rather complex pathway [17,25]. Inactivation of *rirA* in *R. leguminosarum*, *S. meliloti* and *A. tumefaciens* results in differential expression of a battery of genes with the remarkable upregulation of genes involved in iron uptake, whose expression is normally reduced under iron-sufficient conditions [21,22,23,24,26,27]. Our data indicate that *S. fredii* HH103 RirA functions as a regulatory protein. Firstly, like in *R. leguminosarum* and *A. tumefaciens* [21,27], *rirA* transcription in HH103 is subject to autoregulation since transcript levels decreased notably in the complemented strain compared with those observed in the mutant. It should be mentioned that, whereas *rirA* expression remained relatively constant in the complemented strain regardless of the iron levels in the medium, a slight but significant increase in transcriptional activity of *rirA* was detected in the *rirA* null mutant when grown in iron-rich medium compared to iron-deficient medium. This might indicate that *rirA* expression is controlled not only by its own product but also by another iron responsive regulator (e.g., Irr). In fact, a similar expression pattern was described in *A. tumefaciens*, where Irr was shown to repress *rirA* transcription [27]. Secondly, our data show that HH103 RirA not only controls its own expression but also that of the upstream iron-responsive gene *fbpA*, either directly or indirectly. Under iron-sufficient conditions, inactivation of *rirA* in HH103 leads to increased *fbpA* transcript levels compared with those detected in the wild-type. Similar results were obtained in transcriptomic studies performed in *S. meliloti* where the *fbpA* gene was induced under iron-limiting conditions and by *rirA* mutation [23]. Moreover, in silico predictions (RegPrecise (http://regprecise.lbl.gov/RegPrecise/regulog.jsp?regulog_id=1959) [34]) include FbpA as part of the RirA regulon in different rhizobia as well as in *Brucella melitensis*. In HH103, *fbpA* codes for a 347 aa-long protein, which shows significant homology to the ferric ABC transporter substrate-binding protein FbpA that is present in many bacteria, including pathogens [35]. This periplasmic protein participates in iron uptake by shuttling iron from the outer to the inner membrane. Although functional characterization of *fpbA* in HH103 is needed to determine its role in iron uptake, our expression data suggest that, similarly to other *Rhizobiaceae* members, HH103 RirA participates in iron homeostasis.

It is known that RirA regulates gene expression by binding to IRO boxes (5’-TGA-(N9)-TCA-3’) located in the promoter region [14,19,20]. The intergenic *fbpA-rirA* region does not contain any IRO box. The closest IRO box sequence is located 426 nucleotides upstream of the *rirA* start codon and within the *fbpA* coding region. Additional studies are required in order to determine if this sequence or alternative imperfect IRO box sequences that could be present in the intergenic *fbpA-rirA* region might be binding sites for HH103 RirA.

The absence of RirA leads to species-specific growth phenotypes and increased sensitivity to oxidants [21,23,27]. Whereas inactivation of *rirA* in *S. meliloti* results in reduced growth only in iron-rich medium probably as a result of toxic intracellular iron levels, the same mutation in *R. leguminosarum* and *A. tumefaciens* does not cause an iron-dependent growth phenotype, although the *R. leguminosarum rirA* mutant grows slower than the wild-type on all tested media. In *S. fredii* HH103, inactivation of *rirA* leads to a growth advantage in media with low iron content. This phenotype is not caused by constitutive expression of a siderophore, since no CAS activity could be detected in either the wild-type or the *rirA* mutant. In fact, siderophore production seems to be a rare trait among soybean-nodulating rhizobia. *B. japonicum* USDA 110, another soybean symbiont, does not synthesize siderophores [36], and our data revealed that only two out of 40 different *S. fredii* strains tested exhibited CAS activity. Recently, iron availability has been shown to be the main factor influencing the distribution of soybean-nodulating rhizobia in China, with *S. fredii* strains associated with alkaline soils with low iron content, while *Bradyrhizobium* strains are found in soils with high iron content [37]. Therefore, it is tempting to suggest that the increased capacity of the *S. fredii* HH103 *rirA* mutant to grow under iron-limiting conditions might be caused by derepression of as yet unknown high-affinity iron uptake systems. This together with the observed *fbpA* upregulation in the *rirA* mutant, supports the notion that RirA controls iron homeostasis in HH103.

Although a high iron concentration in the medium (220 µM) negatively interferes with *S. fredii* HH103 growth indicating a toxic effect, no differences were detected between the wild-type and the *rirA* mutant. A possible explanation is that HH103 encodes effective systems that protect cells from an iron overload (e.g., iron storage proteins and/or iron efflux transporters), and that are not under RirA control. In this context, the regulation and role in iron homeostasis of *SfHH103_03210* (*bfr*), a gene that putatively codes for bacterioferritin are worth further investigation. In *S. meliloti*, *bfr* expression is enhanced under iron-sufficient conditions and the gene affects the oxidative stress response and symbiotic phenotype of this *Rhizobium* [25]. Another non-excluding possibility that might explain why the *rirA* mutant shows similar resistance to high iron levels as the wild-type implies the existence of additional regulatory control on iron uptake systems in HH103 that function under high iron conditions. While the role of Irr awaits investigation, the participation of the regulatory protein MucR1 in regulating the expression of iron uptake systems (*afuABC*, *hmuS* and *fbpA*) has been reported in *S. fredii* strains [38,39].

Like the *rirA* mutants of *S. meliloti* and *A. tumefaciens*, *S. fredii* HH103 *rirA* is hypersensitive to H_2_O_2_. Hypersensitivity to peroxides in the former mutants was explained by the increased production of highly reactive oxygen radicals resulting from increased intracellular iron levels upon derepression of iron uptake systems. The same rationale could serve to explain why HH103 *rirA* is more sensitive to oxidative stress. The nature of the iron uptake systems that contribute to a metal overload in the absence of RirA have not been investigated in this study. Nevertheless, considering the upregulation of *fbpA* in the mutant, it is reasonable to venture the participation of ferric type ABC transporters. Iron and H_2_O_2_ toxicities are intertwined. However, the behavior of HH103 suggests that the cell damaging effects of H_2_O_2_ are more intense than those caused by the excess of iron. Compared to *S. meliloti* and *A. tumefaciens*, HH103 is much more sensitive to hydrogen peroxide. This fact suggests that HH103 does not possess an efficient antioxidant response.

In contrast to the symbiotic behavior of *rirA* mutants of *R. leguminosarum* and *S. meliloti* with their respective hosts, inactivation of *rirA* in *S. fredii* HH103 impairs the establishment of effective symbiosis with soybean plants, a legume that develops determinate-type nodules. The mutant is able to induce nodule formation on soybean roots but is less efficient in symbiotic nitrogen fixation than the wild-type strain as determined by the reduced nitrogenase activity and plant dry weight. The significant reduction in the number of bacteria present in infected cells in nodules induced by the *rirA* mutant could explain the lower effectiveness of the symbiotic interaction. The reasons for the reduced number of *rirA* cells within soybean nodules are unknown, although it is tempting to speculate that the mutant’s hypersensitivity to oxidants might play a role. During the establishment of the symbiotic process, ROS are generated [40,41,42,43,44,45]. In order to establish a chronic infection within the host, rhizobia need to counteract the plant oxidative burst by using different strategies [44]. Due to the inability to repress iron uptake systems, ROS produced in the symbiotic interaction could have more detrimental effects on a *rirA* mutant than in the wild-type. Nevertheless, it remains to be determined if the role of HH103 RirA in contributing to the development of an efficient symbiosis is exclusive for the interaction with soybean plants, or can be extended to other legume hosts, including those that form indeterminate nodules. In *A. tumefaciens*, RirA is important for tumor formation on tobacco leaf pieces [26] but not on potato disks [27], indicating that the host plays an important role in the outcome of the interaction with mutants lacking this regulatory protein.

In summary, this work represents a basis for the study of iron homeostasis in *S. fredii* HH103. Although investigations on iron homeostasis regulation in members of *Rhizobiaceae* are limited, data obtained indicate that it is a very complex process. This complexity has been highlighted in a recent study performed in *R. leguminosarum* where iron-responsive genes were found to be regulated by O_2_ in an RirA-independent fashion [20]. Despite the complexity of this biological process in which different regulators and signals might be involved, the identification and characterization of the different elements that participate in controlling intracellular iron pools deserves attention as a means to unveil genes that, like RirA, are crucial for the free-living and symbiotic lifestyles of broad host range rhizobia.

## 4. Materials and Methods

### 4.1. Bacterial Strains, Plasmids, and Growth Conditions

Bacterial strains and plasmids used in this study are listed in Table S1. *Escherichia coli* strains were grown in Luria–Bertani (LB) medium [46] at 37 °C; *S. fredii* strains were grown at 28 °C in tryptone-yeast extract (TY) medium [47] or in minimal medium (MM) containing different concentrations of FeCl_3_ [48]. The MM was prepared without iron (un-supplemented) and 100-fold concentrated stock solutions of FeCl_3_ were added to obtain the desired concentration of FeCl_3_.

### 4.2. Construction of the S. fredii rirA Mutant and Complementation.

The *rirA* mutant strain SVQ780 was obtained by allelic exchange. To obtain the mutant allele, a 1391 bp fragment containing the *rirA* gene of *S. fredii* SVQ269, a Rif^R^ derivative of strain HH103, was PCR amplified using primers rirA-F and rirA-R (Table S2), and cloned into pGEM-T-Easy generating plasmid pMUS1265. After DNA sequencing, the *rirA* gene of the construct was disrupted by inserting a cassette into a unique *Bgl*II site located in its coding sequence. For that, the pMUS1265 plasmid was digested with *Bgl*II, treated with Klenow DNA Polymerase to make blunt ends, and ligated to a 4.3 kb *Sma*I fragment containing the *lacZ*-Gm^R^ cassette previously isolated from pAB2001 [49]. The resulting construct, pMUS1276, was digested with *Eco*RI, and the 5.7 kb fragment containing the disrupted *rirA* gene was subcloned into pK18*mobsacB* to yield plasmid pMUS1287. This plasmid was introduced into SVQ269 via conjugation with *E. coli* strain S17-1, and allele replacement events were selected as described previously [50]. Allelic replacement in SVQ780 was confirmed by PCR and DNA-DNA hybridization.

For complementation of the *rirA* mutation present in SVQ780, the wild-type version of *rirA* was PCR amplified as described above and subcloned as an *Eco*RI/*Kpn*I fragment into pK18*mob*. The resulting plasmid, pMUS1406, was conjugated into SVQ780 and Km^R^ transconjugants were selected in order to isolate clones in which plasmid pMUS1406 had been integrated by a single recombination event. One of these transconjugants, named SVQ780C, was selected for further studies. The genomic organization in SVQ780C shown in Figure S3 was determined by PCR.

### 4.3. Siderophore Detection

The determination of siderophores in liquid cultures was performed using the Chrome Azurol S (CAS) assay solution described by Schwyn and Neilands [51]. Supernatants of *S. fredii* cultures grown in un-supplemented MM or MM containing different concentrations of FeCl_3_ were mixed 1:1 with the CAS assay solution. After reaching equilibrium, the absorbance was measured at 630 nm. Alternatively, siderophore production was assessed by the appearance of an orange halo on CAS-agar plates upon contact with bacterial cells previously grown on TY or MM plates.

### 4.4. Sensitivity to H_2_O_2_

Rhizobial cultures were grown in TY medium up to an OD_600nm_ of 1 and subject to different treatments. To determine the rate of survival upon H_2_O_2_ challenge, the cultures were exposed to the desired concentration of H_2_O_2_ (10 mM or 100 µM) for 1 h. The survival rate was calculated by determining the colony forming units (CFU) obtained on TY after plating serial dilutions of non-treated cultures and cultures exposed to H_2_O_2_. To test growth on plates containing H_2_O_2_, rhizobial cells grown in TY broth were washed and resuspended in the same volume of liquid MM. The cell suspension was then tenfold serially diluted and an aliquot (10 µL) of each dilution was spotted onto TY plates containing different concentrations of H_2_O_2_. The experiment was repeated at least twice to ensure the reproducibility of the results.

### 4.5. Reverse Transcription Quantitative PCR (RT-qPCR)

Total RNA was isolated using the High Pure RNA Isolation Kit (Roche, Mannheim, Germany) and RNase Free DNA Set (Qiagen, Hilden, Germany) according to the manufacturer’s instructions. This (DNA free) RNA was reverse transcribed to cDNA by using PrimeScript RT reagent Kit with gDNA Eraser (Takara, Mountain View, CA, USA). Quantitative PCR was performed using a LightCycler 480 (Roche, Basel, Switzerland) with the following conditions: 95 °C, 10 min; 95 °C, 30 s; 50 °C, 30 s; 72 °C, 20 s; forty cycles, followed by the melting curve profile from 60 to 95 °C to verify the specificity of the reaction. The *S. fredii* HH103 16S rRNA was used as an internal control to normalize gene expression. The fold changes of two biological samples with three technical replicates in each condition were obtained using the ∆∆Ct method [52]. Selected genes and primers are listed in Table S2.

### 4.6. Nodulation Assays

Nodulation assays on *Glycine max* (L.) Merr. cv. Williams (soybean) were carried out as previously described [53]. Each Leonard jar contained two plants, and each plant was inoculated with about 5 × 10^8^ bacteria. Plants were grown for 7 weeks in a plant growth chamber with a 16 h photoperiod at 25 °C in the light and 18 °C in the dark. Plant tops were dried at 70 °C for 48 h and weighed. Bacterial isolation from surface-sterilized nodules was carried out as previously described by Buendía-Clavería et al. [54]. Nitrogenase activity of nodules was assessed by ARA as described by Buendía-Clavería and associates [55]. For the different parameters analyzed, the values of each treatment were compared to those of *S. fredii* SVQ269 by using the Mann–Whitney non-parametrical test. At least two independent plant tests were carried out for each strain and host plant. Table 1 shows a representative experiment.

### 4.7. Microscopy Studies

For optical microscopy studies, small fragments of nodules (15–20 days old) were immediately fixed in 4% (*v*/*v*) glutaraldehyde in 0.1 M cacodylate buffer, pH 7.2 for 2 h at 4 °C after collection. Samples were washed several times in 0.1 M cacodylate buffer, pH 7.2, dehydrated in acetone at progressively higher concentrations and embedded in Epon (epoxy embedding medium). The semi-thin sections (0.3–0.5 µm) were obtained on a Reichert–Jung Ultracut E ultramicrotome, stained with Toluidine blue and viewed in a Leitz (Aristoplan) light microscope (Leica, Munich, Germany).

## Figures and Tables

**Table 1:** Symbiotic capacity of *S. fredii* SVQ269, its *rirA* (SVQ780) mutant derivative and the complemented version (SVQ780C) with *Glycine max* cv. Williams.

*Glycine max* cv.Williams ^1^					
Inoculant	Number of Nodules	Nodules Fresh Weight (mg)	Plant-Top Dry Weight (mg)	ARA ^2^ (Nmoles Acetylene/Plant/Hour)	Nodules Size (mm)
SVQ269	26.38 ± 2.88	510.33 ± 65.71	1.08 ± 0.15	998.66 ± 175.56	4.04 ± 0.11
SVQ780	23.80 ± 3.38	222.71 ± 16.04 **	0.62 ± 0.07 *	167.99 ± 27.64 **	2.64 ± 0.10 **
SVQ780C	22.44 ± 2.50	453.48 ± 54.89	1.18 ± 0.12	1008.22 ± 118.18	4.37 ± 0.10 **
NI ^3^	0	0	0.245 ± 0.02 **	0	0

^1^ Numbers are mean (± standard error of the mean, SEM) values per plant. Ten plants were tested for each *G. max*/inoculant combination. Plants were grown for six weeks in a plant growth chamber. All the treatments were individually compared with the values of the parental strain, using the nonparametric test of Mann–Whitney. The presence of one or two asterisks denotes a significant difference with respect to data obtained in plants inoculated with the wild-type (*p* value < 0.05 or < 0.01, respectively). ^2^ Acetylene Reduction Assays (ARA). ^3^ Non-Inoculated (NI).

## Supplementary Materials

Supplementary materials can be found at http://www.mdpi.com/1422-0067/20/3/787/s1.

## References

[1] Andrews S.C., Robinson A.K., Rodríguez-Quiñones F. (2003). Bacterial iron homeostasis. FEMS Microbiol. Rev..

[2] Imlay J.A. (2003). Pathways of oxidative damage. Annu. Rev. Microbiol..

[3] Imlay J.A. (2014). The mismetallation of enzymes during oxidative stress. J. Biol. Chem..

[4] Saha R., Saha N., Donofrio R.S., Bestervelt L.L. (2013). Microbial siderophores: A mini review. J. Basic Microbiol..

[5] Frawley E.R., Fang F.C. (2014). The ins and outs of bacterial iron metabolism. Mol. Microbiol..

[6] Chandrangsu P., Rensing C., Helmann J.D. (2017). Metal homeostasis and resistance in bacteria. Nat. Rev. Microbiol..

[7] Pi H., Helmann J.D. (2017). Ferrous iron efflux systems in bacteria. Metallomics.

[8] Hantke K. (1981). Regulation of ferric iron transport in *Escherichia coli* K12: Isolation of a constitutive mutant. Mol. Gen. Genet..

[9] Bsat N., Herbig A., Casillas-Martinez L., Setlow P., Helmann J.D. (1998). *Bacillus subtilis* contains multiple Fur homologues: Identification of the iron uptake (Fur) and peroxide regulon (PerR) repressors. Mol. Microbiol..

[10] Fleischhacker A.S., Kiley P.J. (2011). Iron-containing transcription factors and their roles as sensors. Curr. Opin. Chem. Biol..

[11] Poole P., Ramachandran V., Terpolilli J. (2018). Rhizobia: From saprophytes to endosymbionts. Nat. Rev. Microbiol..

[12] Chao T.C., Becker A., Buhrmester J., Pühler A., Weidner S. (2004). The *Sinorhizobium meliloti fur* gene regulates, with dependence on Mn(II), transcription of the *sitABCD* operon, encoding a metal-type transporter. J. Bacteriol..

[13] Diaz-Mireles E., Wexler M., Sawers G., Bellini D., Todd J.D., Johnston A.W. (2004). The Fur-like protein Mur of *Rhizobium leguminosarum* is a Mn(2+)-responsive transcriptional regulator. Microbiology.

[14] Rodionov D.A., Gelfand M.S., Todd J.D., Curson A.R., Johnston A.W. (2006). Computational reconstruction of iron- and manganese-responsive transcriptional networks in alpha-proteobacteria. PLoS Comput. Biol..

[15] Rudolph G., Hennecke H., Fischer H.M. (2006). Beyond the Fur paradigm: Iron-controlled gene expression in rhizobia. FEMS Microbiol. Rev..

[16] Johnston A.W., Todd J.D., Curson A.R., Lei S., Nikolaidou-Katsaridou N., Gelfand M.S., Rodionov D.A. (2007). Living without Fur: The subtlety and complexity of iron-responsive gene regulation in the symbiotic bacterium *Rhizobium* and other alpha-proteobacteria. Biometals.

[17] O’Brian M.R. (2015). Perception and homeostatic control of iron in the Rhizobia and related bacteria. Annu. Rev. Microbiol..

[18] Hamza I., Chauhan S., Hassett R., O’Brian M.R. (1998). The bacterial Irr protein is required for coordination of heme biosynthesis with iron availability. J. Biol. Chem..

[19] Yeoman K.H., Curson A.R., Todd J.D., Sawers G., Johnston A.W. (2004). Evidence that the *Rhizobium* regulatory protein RirA binds to *cis*-acting iron-responsive operators (IROs) at promoters of some Fe-regulated genes. Microbiology.

[20] Pellicer Martinez M.T., Martinez A.B., Crack J.C., Holmes J.D., Svistunenko D.A., Johnston A.W.B., Cheesman M.R., Todd J.D., Le Brun N.E. (2017). Sensing iron availability via the fragile [4Fe-4S] cluster of the bacterial transcriptional repressor RirA. Chem. Sci..

[21] Todd J.D., Wexler M., Sawers G., Yeoman K.H., Poole P.S., Johnston A.W. (2002). RirA, an iron-responsive regulator in the symbiotic bacterium *Rhizobium leguminosarum*. Microbiology.

[22] Todd J.D., Sawers G., Johnston A.W. (2005). Proteomic analysis reveals the wide-ranging effects of the novel, iron-responsive regulator RirA in *Rhizobium leguminosarum* bv. *viciae*. Mol. Genet. Genom..

[23] Chao T.C., Buhrmester J., Hansmeier N., Pühler A., Weidner S. (2005). Role of the regulatory gene *rirA* in the transcriptional response of *Sinorhizobium meliloti* to iron limitation. Appl. Environ. Microbiol..

[24] Viguier C., Cuiv O., Clarke P., O’Connell M. (2005). RirA is the iron response regulator of the rhizobactin 1021 biosynthesis and transport genes in *Sinorhizobium meliloti* 2011. FEMS Microbiol. Lett..

[25] Costa D., Amarelle V., Valverde C., O’Brian M.R., Fabiano E. (2017). The Irr and RirA proteins participate in a complex regulatory circuit and act in concert to modulate bacterioferritin expression in *Ensifer meliloti* 1021. Appl. Environ. Microbiol..

[26] Ngok-Ngam P., Ruangkiattikul N., Mahavihakanont A., Virgem S.S., Sukchawalit R., Mongkolsuk S. (2009). Roles of *Agrobacterium tumefaciens* RirA in iron regulation, oxidative stress response, and virulence. J. Bacteriol..

[27] Hibbing M.E., Fuqua C. (2011). Antiparallel and interlinked control of cellular iron levels by the Irr and RirA regulators of *Agrobacterium tumefaciens*. J. Bacteriol..

[28] Dokpikul T., Chaoprasid P., Saninjuk K., Sirirakphaisarn S., Johnrod J., Nookabkaew S., Sukchawalit R., Mongkolsuk S. (2016). Regulation of the Cobalt/Nickel Efflux Operon *dmeRF* in *Agrobacterium tumefaciens* and a Link between the Iron-Sensing Regulator RirA and Cobalt/Nickel Resistance. Appl. Environ. Microbiol..

[29] Margaret I., Becker A., Blom J., Bonilla I., Goesmann A., Gottfert M., Lloret J., Mittard-Runte V., Ruckert C., Ruiz-Sainz J.E. (2011). Symbiotic properties and first analyses of the genomic sequence of the fast growing model strain *Sinorhizobium fredii* HH103 nodulating soybean. J. Biotechnol..

[30] López-Baena F.J., Ruiz-Sainz J.E., Rodríguez-Carvajal M.A., Vinardell J.M. (2016). Bacterial Molecular Signals in the *Sinorhizobium fredii*-Soybean Symbiosis. Int. J. Mol. Sci..

[31] Weidner S., Becker A., Bonilla I., Jaenicke S., Lloret J., Margaret I., Puhler A., Ruiz-Sainz J.E., Schneiker-Bekel S., Szczepanowski R. (2012). Genome sequence of the soybean symbiont *Sinorhizobium fredii* HH103. J. Bacteriol..

[32] Vinardell J.M., Acosta-Jurado S., Zehner S., Gottfert M., Becker A., Baena I., Blom J., Crespo-Rivas J.C., Goesmann A., Jaenicke S. (2015). The *Sinorhizobium fredii* HH103 genome: A comparative analysis with *S. fredii* strains differing in their symbiotic behavior with soybean. Mol. Plant Microbe Interact..

[33] Carter R.A., Yeoman K.H., Klein A., Hosie A.H., Sawers G., Poole P.S., Johnston A.W. (2002). *dpp* genes of *Rhizobium leguminosarum* specify uptake of delta-aminolevulinic acid. Mol. Plant Microbe Interact..

[34] Novichkov P.S., Kazakov A.E., Ravcheev D.A., Leyn S.A., Kovaleva G.Y., Sutormin R.A., Kazanov M.D., Riehl W., Arkin A.P., Dubchak I. (2013). RegPrecise 3.0—a resource for genome-scale exploration of transcriptional regulation in bacteria. BMC Genom..

[35] Parker Siburt C.J., Mietzner T.A., Crumbliss A.L. (2012). FbpA--a bacterial transferrin with more to offer. Biochim. Biophys. Acta.

[36] Guerinot M.L., Meidl E.J., Plessner O. (1990). Citrate as a siderophore in *Bradyrhizobium japonicum*. J. Bacteriol..

[37] Yang S.H., Chen W.H., Wang E.T., Chen W.F., Yan J., Han X.Z., Tian C.F., Sui X.H., Singh R.P., Jiang G.M. (2018). Rhizobial biogeography and inoculation application to soybean in four regions across China. J. Appl. Microbiol..

[38] Jiao J., Wu L.J., Zhang B., Hu Y., Li Y., Zhang X.X., Guo H.J., Liu L.X., Chen W.X., Zhang Z. (2016). MucR is required for transcriptional activation of conserved ion transporters to support nitrogen fixation of *Sinorhizobium fredii* in soybean nodules. Mol. Plant Microbe Interact..

[39] Acosta-Jurado S., Alias-Villegas C., Navarro-Gómez P., Zehner S., Murdoch P.D., Rodríguez-Carvajal M.A., Soto M.J., Ollero F.J., Ruiz-Sainz J.E., Gottfert M. (2016). The *Sinorhizobium fredii* HH103 MucR1 global regulator is connected with the *nod* regulon and is required for efficient symbiosis with *Lotus burttii* and *Glycine max* cv. Williams. Mol. Plant Microbe Interact..

[40] Santos R., Herouart D., Sigaud S., Touati D., Puppo A. (2001). Oxidative burst in alfalfa-*Sinorhizobium meliloti* symbiotic interaction. Mol. Plant Microbe Interact..

[41] Bueno P., Soto M.J., Rodríguez-Rosales M.P., Sanjuan J., Olivares J., Donaire J.P. (2001). Time-course of lipoxygenase, antioxidant enzyme activities and H_2_O_2_ accumulation during the early stages of *Rhizobium*-legume symbiosis. New Phytol..

[42] Ramu S.K., Peng H.M., Cook D.R. (2002). Nod factor induction of reactive oxygen species production is correlated with expression of the early nodulin gene *rip1* in *Medicago truncatula*. Mol. Plant Microbe Interact..

[43] Shaw S.L., Long S.R. (2003). Nod factor inhibition of reactive oxygen efflux in a host legume. Plant Physiol..

[44] Chang C., Damiani I., Puppo A., Frendo P. (2009). Redox changes during the legume-rhizobium symbiosis. Mol. Plant.

[45] Damiani I., Pauly N., Puppo A., Brouquisse R., Boscari A. (2016). Reactive oxygen species and nitric oxide control early steps of the legume-*Rhizobium* symbiotic interaction. Front. Plant Sci..

[46] Sambrook J., Fritsch E.F., Maniatis T. (1989). Molecular Cloning: A Laboratory Manual.

[47] Beringer J.E. (1974). R factor transfer in *Rhizobium leguminosarum*. J. Gen. Microbiol..

[48] Nogales J., Domínguez-Ferreras A., Amaya-Gómez C.V., van Dillewijn P., Cuéllar V., Sanjuán J., Olivares J., Soto M.J. (2010). Transcriptome profiling of a *Sinorhizobium meliloti fadD* mutant reveals the role of rhizobactin 1021 biosynthesis and regulation genes in the control of swarming. BMC Genom..

[49] Becker A., Schmidt M., Jäger W., Pühler A. (1995). New gentamicin-resistance and *lacZ* promoter-probe cassettes suitable for insertion mutagenesis and generation of transcriptional fusions. Gene.

[50] Schäfer A., Tauch A., Jager W., Kalinowski J., Thierbach G., Pühler A. (1994). Small mobilizable multi-purpose cloning vectors derived from the *Escherichia coli* plasmids pK18 and pK19: Selection of defined deletions in the chromosome of *Corynebacterium glutamicum*. Gene.

[51] Schwyn B., Neilands J.B. (1987). Universal chemical assay for the detection and determination of siderophores. Anal. Biochem..

[52] Pfaffl M.W. (2001). A new mathematical model for relative quantification in real-time RT-PCR. Nucleic Acids Res..

[53] Hidalgo A., Margaret I., Crespo-Rivas J.C., Parada M., Murdoch Pdel S., López A., Buendía-Clavería A.M., Moreno J., Albareda M., Gil-Serrano A.M. (2010). The *rkpU* gene of *Sinorhizobium fredii* HH103 is required for bacterial K-antigen polysaccharide production and for efficient nodulation with soybean but not with cowpea. Microbiology.

[54] Buendía-Clavería A.M., Moussaid A., Ollero F.J., Vinardell J.M., Torres A., Moreno J., Gil-Serrano A.M., Rodríguez-Carvajal M.A., Tejero-Mateo P., Peart J.L. (2003). A *purL* mutant of *Sinorhizobium fredii* HH103 is symbiotically defective and altered in its lipopolysaccharide. Microbiology.

[55] Buendía-Clavería A.M., Chamber M., Ruiz-Sainz J.E. (1989). A comparative study of the physiological characteristics, plasmid content and symbiotic properties of different *Rhizobium fredii* strains. Syst. Appl. Microbiol..

