# Supplementary material for: Sinorhizobium fredii HH103 RirA Is Required for Oxidative Stress Resistance and Efficient Symbiosis with Soybean
Source: Int J Mol Sci. 2019 Feb 12;20(3):787. doi: 10.3390/ijms20030787 (PMC6386902; doi:10.3390/ijms20030787)
Supplement: Supplementary file 1 [file ijms-20-00787-s001.pdf]

Supplementary Materials

Sinorhizobium fredii HH103 RirA is required for oxidative stress resistance and efficient symbiosis with soybean

Juan Carlos Crespo-Rivas [1,†], Pilar Navarro-Gómez [1,†], Cynthia Alias-Villegas [1,†], Jie Shi [2], Tao Zhen [3], Yanbo Niu [3], Virginia Cuéllar [4], Javier Moreno [5], Teresa Cubo [1], José María Vinardell [1], José Enrique Ruiz-Sainz [1], Sebastián Acosta-Jurado [1,*] and María José Soto [4,*]

```
                                                     ★        ★
At       (1) MRLTKQTNYAVRMLMYCAANEGKLSRIPEIARAYGVSELFLFKILQPLNK
Rl       (1) MRLTKQTNYAVRMLMYCAANDGHLSRIPEIAKAYGVSELFLFKILQPLNK
Sm       (1) MRLTKQTNYAVRMLMYCAANGEKLSRIPEIARAYGVSELFLFKILQPLTR
SfNGR    (1) MRLTKQTNYAVRMLMYCAANGEKLSRIPEIAKAYGVSELFLFKILQPLTR
SfHH     (1) MRLTKQTNYAVRMLMYCAANGEKLSRIPEIAKAYGVSELFLFKILQPLTR

At      (51) AGLVETVRGRNGGVRLGKPAEKISLFDVVKVTEDSFAMAECFEDG-AVEC
Rl      (51) AGLVETVRGRNGGVRLGKPAADISLFDVVRVTEDSFAMAECFEDDGEVEC
Sm      (51) AGLVETVRGRNGGVRLPRPASEITLFDVVKVTEDSFAMAECFEAG-EIDC
SfNGR   (51) AGLVETVRGRNGGVRLPKPASEISLFDVVKVTEDSFAMAECFEAG-EIDC
SfHH    (51) AGLVETVRGRNGGVRLPKPASEISLFDVVKVTEDSFAMAECFEAG-EIDC

                  ★
At     (100) PLVDSCGLNSALRKALNAFFDVLAEYSIDDLVKARPQINFLLGIDTEMPKIAALPAA--- (156)
Rl     (101) PLVDSCGLNSALRKALNAFFAVLSEYSIDDLVKARPQINFLLGITGEPAYRKPAIVAPAA (160)
Sm     (100) PLVDSCGLNAALRKALNAFFEVLQGYTIDDLVKARPQINFLLGLEEPVRPQTSAA----- (154)
SfNGR  (100) PLVDSCGLNAALRKALNAFFDVLKGYTIDDLVKARPQINFLLGLETGPRPQTSAA----- (154)
SfHH   (100) PLVDSCGLNAALRKALNAFFDVLKGYSIDDLVKARPQINFLLGLDTGPRPQTSAA----- (154)
```

Figure S1. Alignment of RirA from *Sinorhizobium fredii* HH103 (SfHH, Accession Number WP_014327406.1) with RirA sequences from *Agrobacterium tumefaciens* (At, Accession Number WP_003514531*),* *Rhizobium leguminosarum* (Rl, Accession Number WP_003545647), *Sinorhizobium meliloti* (Sm, Accession Number WP_003527122), and *S. fredii* NGR234 (SfNGR, Accession Number WP_012706927.1). Identical residues are indicated in red and highlighted in yellow. Conservative and similar residues are indicated in light blue and green backgrounds, respectively. Red stars indicate the three conserved cysteine residues predicted to ligate iron–sulfur clusters in Rrf2 family regulators.

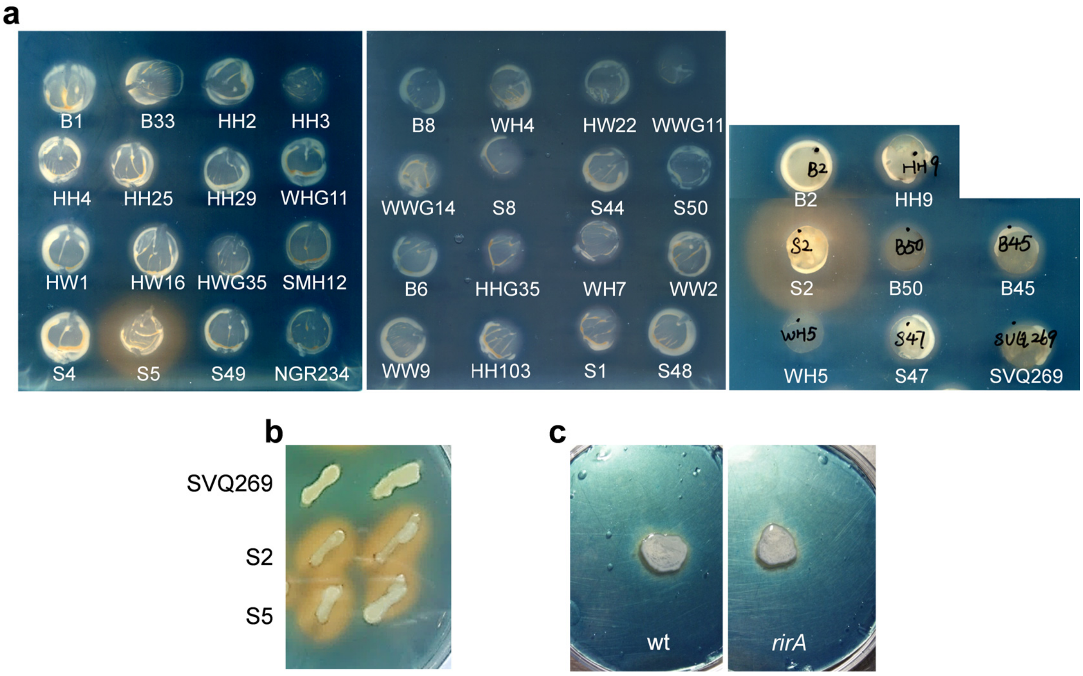

Figure S2. Siderophore production in *S. fredii* strains. CAS activity observed on CAS-agar plates after incubating *S. fredii* cells previously grown on TY plates (a) or on MM containing 22 µM $FeCl_3$ (b) and (c). Only strains S2 and S5 exhibited orange halos on CAS agar plates indicative of siderophore production. Pictures were taken 3-4 days (a and b) or 28 days (c) after incubation of cells in the CAS-agar. In (c) wt and *rirA* **i**ndicates *S. fredii* wild-type strain SVQ269 and its *rirA* derivative mutant SVQ780.

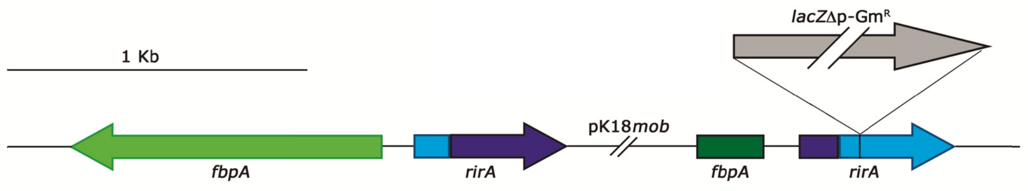

Figure S3. Genomic organisation of the complemented strain SVQ780C. The *rirA* and *fbpA* sequences corresponding to the pMUS1406 construct are indicated in purple and dark green, respectively.

Table S1. Bacterial strains and plasmids used in this study.

Strain/plasmid	Relevant characteristics[a]	Reference/source
**Strains**		
Sinorhizobium fredii		
SVQ269	Spontaneous Rif^R derivative of HH103; Rif^R	[1]
SVQ780	SVQ269 *rirA*::l*ac*ZΔp-Gm; Rif^R, Gm^R	This work
SVQ780C	SVQ780 derivative with pMUS1406 co-integrated; Rif^R, Gm^R, Km^R	This work
B1		
B2		
B6		
B8	Strains isolated from Xinjian Autonomous Region (Wester China)	
B33		
B45		
B50		
HW1		
HW16		
HW22		
HWG35		
WW2	Strains isolated from Shandong (East-Central coastal China)	
WW9		
WWG11		
WWG14		
HH2		
HH3		
HH4		[2]
HH9		
HH25		
HH29	Strains isolated from Henan (Central China)	
HHG35		
WH4		
WH5		
WH7		
WHG11		
S1		
S2		
S4		
S5		
S8		
S44	Strains isolated from Hubei (Central China)	
S47		
S48		
S49		
S50		
HH103	Wild type strain	[3]
SMH12	Wild type strain, Ap^R	[4]

NGR234	*S. fredii* strain isolated from nodules of *Lablab purpureus*, Rif^R	[5]
Escherichia coli		
DH5α	*supE44, DlacU169, f80, lacZDM, 5hsdR171, recA1, endA1, gyrA96, thi-1, relA1*	Bethesda Research Lab
S17-1	*thi, pro, recA, hsdR, hsdM*, RP4-2-Tc::Mu-Km::Tn7	[6]
**Plasmids**		
pGEM-T	Cloning vector; Ap^R	Promega
pAB2001	Plasmid containing the l*acZ*Δp-Gm cassette; Ap^R, Gm^R	[7]
pK18*mob*/ pK18*mobsacB*	Suicide vectors, Km^R	[8]
pMUS1265	pGEM-T derivative containing HH103 *rirA*; Ap^R	This work
pMUS1276	pMUS1265 derivative containing *rirA*::l*acZ*Δp-Gm; Ap^R, Gm^R	This work
pMUS1287	pK18*mobsacB* derivative containing *rirA*::l*acZ*Δp-Gm; Km^R, Gm^R	This work
pMUS1406	pK18*mob* derivative containing HH103 *rirA*; Km^R	This work

[a] Rif^R, Gm^R, Km^R, and Ap^R, indicate rifampicin, gentamicin, kanamycin, and ampicillin resistance, respectively.

Table S2. Primers used in polymerase chain reaction (PCR) experiments and quantitative PCR (*q*PCR).

Primer	Sequence (5′-3′)	Predicted length of PCR products (bp)	Use
rirA-F	TGTCGACGGTCAGAATGACG	1391	Mutagenesis of *rirA*
rirA-R	ACGGTTATAGACGGTGTGCG		
lacZintR	GCCTCTTCGCTATTACGCCA	-	Checking *rirA* mutant and complemented strains
qrirAF	AGATCGCCAAGGCTTACG	148	*q*PCR
qrirAR	ACGTCGAAGAGGCTGATC		
qhmuSF	CCTATGACAGGATTGTCG	163	*q*PCR
qhmuSR	ATGCCGTGGAACTGATGG		
qsufBF	CTATCAGCGTTGGCTCAC	156	*q*PCR
qsufBR	TCATAGACCTTGAGCAGC		
qirrF	TCGAGGAACTGCATGAAG	154	*q*PCR
qirrR	TGATCCGAGACATTGGTG		
rt16S-F2	GATACCCTGGTAGTCCAC	167	*q*PCR
rt16S-R2	TAAACCACATGCTCCACC		
qfbpA F	CACCTACGAGGAACTTGC	159	*q*PCR
qfbpA R	TTGGAGAGATTGTCCCTG		

References

1. Madinabeitia, N.; Bellogín, R.A.; Buendía-Clavería, A.M.; Camacho, M.; Cubo, T.; Espuny, M.R.; Gil-Serrano, A.M.; Lyra, M.C.; Moussaid, A.; Ollero, F.J.; Soria-Díaz, M.E.; Vinardell, J.M.; Zeng, J.; Ruiz-Sainz, J.E. *Sinorhizobium fredii* HH103 has a truncated *nolO* gene due to a -1 frameshift mutation that is conserved among other geographically distant *S. fredii* strains. *Mol Plant Microbe Interact* **2002**, 15, 150-159, DOI: 10.1094/MPMI.2002.15.2.150.
2. Thomas-Oates.; J.; Bereszczak.; J.; Edwards.; E.; Gill.; A.; Noreen S.; Zhou, J.C.; Chen, MZ.; Miao, L.H.; Xie, F.L.; Yang, J.K.; Zhou, Q.; Yang, S.S.; Li, X.H.; Wang, L.; Spaink, H.P.; Schlaman, H.R.; Harteveld, M.; Díaz, C.L.; van Brussel, A.A.; Camacho, M.; Rodríguez-Navarro, DN.; Santamaría, C.; Temprano, F.; Acebes, J.M.; Bellogín, R.A.; Buendía-Clavería, A.M.; Cubo, M.T.; Espuny, M.R.; Gil, A.M.; Gutiérrez, R.; Hidalgo, A.; López-Baena, F.J.; Madinabeitia, N.; Medina, C.; Ollero, F.J.; Vinardell, J.M.; Ruiz-Sainz, J.E. A catalogue of molecular, physiological and symbiotic properties of soybean-nodulating rhizobial strains from different soybean cropping areas of China. *Syst Appl Microbiol* **2003**, 26, 453-65, DOI: 10.1078/072320203322497491.
3. Dowdle, S.F., and Bohlool, B. B. Intra- and inter-specific competition in *Rhizobium fredii* and *Bradyrhizobium japonicum* as indigenous and introduced organisms. *Can J Microbiol* **1987**, 33, 990-995.
4. Rodríguez-Navarro, D.N.; Ruiz-Sainz, J.E.; Buendía-Clavería, A.M.; Santamaría, C.; Balatti, P.A.; Krishnan, H.B.; Pueppke, S.G. Characterization of Fast-Growing Rhizobia from Nodulated Soybean [*Glycine max* (L.) Merr.] in Vietnam. *Syst Appl Microbiol* **1996**, 19, 240-248, DOI: 10.1016/S0723-2020(96)80050-6.
5. Trinick, M.J. Relationships amongst the fast-growing *Rhizobium* of *Lablab purpureus*, *Leucarena leucocephala*, *Mimosa* sp., *Acacia farnesiana*, and *Sesbania grandiflora* and their affinities with other *Rhizobium* groups. *J Appl Bacteriol* **1980**, 49, 39-53, DOI: 10.1111/j.1365-2672.1980.tb01042.x.

6. Simon, R.; Priefer, U.; Pühler, A. A broad host range mobilization system for *in vivo* genetic engineering: Transposon mutagenesis in Gram-negative bacteria. *Nat Biotechnol* **1983**, 1, 784-791.
7. Becker, A.; Schmidt, M.; Jäger, W.; Pühler, A. New gentamicin-resistance and *lacZ* promoter-probe cassettes suitable for insertion mutagenesis and generation of transcripcional fusions. *Gene* **1995**, 162, 37-39.
8. Schäfer, A.; Tauch, A.; Jäger, W.; Kalinowski, J.; Thierbach, G.; Pühler, A. Small mobilizable multi-purpose cloning vectors derived from the *Escherichia coli* plasmids pK18 and pK19: selection of defined deletions in the chromosome of *Corynebacterium glutamicum*. *Gene* **1994**, 145, 69-73.
